# GBR-DETR: A Real-Time Tomato Leaf Disease Detection Model for Edge Device Deployment

**DOI:** 10.3390/s26102950

**Published:** 2026-05-08

**Authors:** Jiaxiong Zhuo, Guikun Dong, Qingfeng Huang, Lei Zhou, Feixiong Zhao, Ping Yuan, Xiangjun Yang

**Affiliations:** School of Mechanical Engineering, Chengdu University, Chengdu 610106, China

**Keywords:** tomato leaf diseases, object detection, RT-DETR, Bidirectional Context Pyramid Network, Jetson Orin Nano

## Abstract

Tomato leaf diseases pose significant threats to crop yield and food security. However, in real-world cultivation environments, factors such as fluctuating illumination, varying leaf occlusion, and ambiguous lesion morphology often compromise detection accuracy. This paper presents the Gradient-aware Bidirectional Retentive Detection Transformer (GBR-DETR), a model designed for high-precision, real-time disease detection. This model is composed of two network structures and a retentive feature aggregation module: (1) a Multi-scale Gradient-Aware Transfer Network (MGAT-Net) is designed to encode gradient information through the Sobel operator, thereby enhancing the localization stability for small and blurry lesions; (2) a Bidirectional Context Pyramid Network (BCPN) is proposed to enable bidirectional interactions among multi-level features through a top-down and a bottom-up pathway, thereby generating multi-scale lesion features and bridging cross-scale semantic gaps; and (3) a Retentive Feature Aggregation Module (RFAM) is used to suppress background noise and establish global feature correlations, thereby enhancing the overall representation capability for lesion recognition. Experiments on the Multi-scenario Tomato Leaf Disease (M-TLD) dataset show that GBR-DETR yields gains of 3.12, 4.88, and 3.41 percentage points in mAP_50–95_, mAP_50_, and mAP_75_, respectively, over the baseline RT-DETR, while also outperforming representative DETR-based and CNN-based detectors. The model demonstrates robust generalization on the PlantDoc cross-domain benchmark, achieving a 2.11% improvement in mAP_50_ over the baseline. Deployed on the NVIDIA Jetson Orin Nano with TensorRT FP16, it achieves 54 ms latency, enabling real-time disease monitoring on edge devices. This solution provides effective technical support for real-time disease monitoring in smart agriculture.

## 1. Introduction

Tomato (*Solanum lycopersicum* L.) is one of the most economically valuable vegetable crops in the world, and it is produced in more than 186 million metric tonnes annually [1]. However, it is significantly affected by foliar diseases caused by fungi, bacteria, and viruses, which can reduce tomato yield by 20–40 percent under epidemic conditions [2]. Early and precise disease diagnosis is thus necessary to protect crops and ensure food security [3]. Classical disease identification methods rely primarily on visual inspection by trained agronomists, an approach that is inherently labor-intensive, subjective, and unable to meet the demands of large-scale, real-time monitoring in modern agricultural systems [4].

The interplay between deep learning and computer vision has created significant opportunities for automated crop disease diagnosis in precision agriculture. Object detection is a fundamental task in computer vision, involving the localization and classification of multiple targets in an image, and is the basis of intelligent visual perception in the agricultural field [5]. The rapid development of detection architectures, including convolutional neural network (CNN)-based detectors (e.g., the YOLO family) and Transformer-based detectors (e.g., the Detection Transformer [DETR]), has significantly enhanced detection accuracy and robustness [6]. These advancements have opened significant opportunities for automated disease surveillance systems in complex field settings. However, CNN-based detectors inherently struggle to capture long-range spatial dependencies and global context, which are crucial for the effective localization of morphologically ambiguous or partially covered disease lesions [3]. Moreover, several recent studies have shown that most current detection methods remain confined to controlled laboratory settings. Little research has systematically examined whether these methods can achieve real-time inference and generalize effectively when deployed on resource-constrained edge devices [7,8,9]. This observation highlights a critical gap between reported detection performance and the operational requirements of practical agricultural monitoring systems.

Great progress has been made in research on the use of deep learning for plant disease recognition. The classification models built with CNNs have demonstrated high accuracy exceeding 95 percent on standard plant disease datasets [10]. In addition to classification, object detection models, specifically the YOLO series, have expanded the ability to localize and identify diseases simultaneously. As an example, Abulizi et al. [11] propose DM-YOLO, which uses YOLOv9 and employs multi-kernel depthwise convolution and dynamic upsampling to enhance the detection of tomato leaf diseases in natural environments. Chen et al. [12] adopt a better YOLOv8n that includes SimAM attention and improved feature pyramid structures, yielding considerable benefits for multi-scale lesion detection. Shen et al. [13] also demonstrate that optimized YOLOv8 architectures based on depthwise grouped convolutions can provide real-time, accurate tomato disease detection for use in sustainable agriculture. These YOLO-based advances, however, largely focus on local feature enhancement within individual scales and do not explicitly address global inter-scale semantic consistency or the preservation of gradient-level boundary cues throughout hierarchical feature propagation [14,15]. As a result, detection performance degrades significantly for early-stage lesions with diffuse margins and low foreground–background contrast, which require holistic contextual reasoning beyond the capabilities of pure convolutional models [16].

On the Transformer side, DETR redefines object detectors as a direct set prediction task and removes candidate-specific components (e.g., anchor boxes and non-maximum suppression (NMS)) using bipartite matching. On the same basis, Real-Time Detection Transformer (RT-DETR) is the first real-time end-to-end Transformer detector, using a hybrid CNN–Transformer encoder and achieving a good speed–accuracy trade-off. Zhang et al. [17] propose WMC-RTDETR for tea disease detection, demonstrating that wavelet-transform convolution and multiscale, multihead self-attention significantly enhance RT-DETR’s lesion-detection capability while reducing the model’s parameter count. Sun et al. [18] propose Eggplant-DETR, which combines multi-scale feature boosting and frequency-domain perception within the RT-DETR platform, achieving state-of-the-art results under complex agricultural conditions. Collectively, these findings suggest that the RT-DETR paradigm remains promising for agricultural disease detection. However, there is still a lack of knowledge about its application to tomato leaf diseases. Transformer architectures are more effective at global modeling, but their quadratic computational cost with respect to input resolution makes them impractical in resource-constrained settings [5]. Nevertheless, existing RT-DETR adaptations for crop disease detection typically address only one of several critical aspects—cross-scale semantic alignment, gradient-aware boundary localization, or background noise suppression—and rarely integrate them optimally within a unified pipeline [19,20]. There is also a dearth of systematic validation on resource-constrained edge devices; thus, the applicability of these techniques in real-world settings is limited [21].

In parallel, multi-scale feature fusion remains a critical challenge in detecting disease lesions of varying sizes. Zheng et al. [22] adopt the traditional PANet feature pyramid architecture, following chain-based fusion steps that gradually smooth out dense pathological features as they are downsampled. Recent literature has examined bidirectional and attention-directed fusion techniques to bridge cross-scale semantic gaps. However, how to simultaneously retain high-resolution spatial details and high-level semantic information in fine-grained agricultural disease detection remains an open problem. In terms of deployment, Nyakuri et al. [23] demonstrate that lightweight CNN models running on a Raspberry Pi-based edge device can detect pests and diseases in real time, with inference latency below 80 ms, highlighting the viability of agricultural intelligence at the device level. However, most existing high-performance detection models lack systematic validation for edge deployment in real-world agricultural environments with limited network connectivity [23].

Consequently, a single large-scale detection system that can provide high accuracy, cross-domain generalization, and edge-deployable efficiency for detecting tomato leaf disease remains an urgent and unsolved research problem. To overcome these drawbacks, a tomato leaf disease detection model—Gradient-aware Bidirectional Retentive Detection Transformer (GBR-DETR)—is proposed. The proposed model incorporates two network structures and a feature aggregation module: gradient-aware backbone enhancement (G), bidirectional feature pyramid fusion (B), and retentive feature aggregation (R). The principal value of this work is as follows:

(1) The paper presents a multi-scenario tomato leaf disease dataset (M-TLD) that uniformly examines changes in illumination, occlusion, planting density, and complexity of the background, as a comprehensive benchmark for assessing detection robustness under conditions of natural agricultural fields.
(2) GBR-DETR integrates three innovative components: the Multi-scale Gradient-Aware Transfer Network (MGAT-Net), which explicitly encodes gradient cues via Sobel operators to enhance localization stability for minute or blurred lesions with linear computational complexity; the Bidirectional Context Pyramid Network (BCPN), which creates multi-scale lesion representations by generating multi-scale information flow through bilateral deep and lateral gradient cues to bridge cross-scale semantic differences; and the Retentive Feature Aggregation Module (RFAM), which filters background noise and establishes global correlations through a retention mechanism with linear complexity, thereby preserving semantic coherence across overlapping lesions.
(3) The engineering feasibility of GBR-DETR is validated through real-field deployment on the NVIDIA Jetson Orin Nano platform, achieving 54 ms of per-frame inference latency with TensorRT FP16 optimization. Experimental findings indicate that GBR-DETR attains 78.91% mAP_50_, 68.52% mAP_75_, and 62.98% mAP_50–95_ on the M-TLD dataset, significantly outperforming baseline CNN- and Transformer-based models, and demonstrating strong cross-domain generalization on the PlantDoc benchmark.

## 2. Materials

### 2.1. Data Collection

The tomato leaf disease image dataset created in this research, hereafter referred to as the Multi-scenario Tomato Leaf Disease dataset (M-TLD), was obtained from various tomato orchards and greenhouse gardens around Chengdu University in Chengdu, Sichuan Province, China. The area has a subtropical humid monsoon climate, with an average annual temperature of about 16.8 °C. The high levels of humidity and rainfall in the area not only provide ideal conditions for tomato plant growth but also lead to the prevalence of foliar diseases. To ensure dataset representativeness and diversity, data were collected in three common cultivation settings: open-field cultivation, plastic tunnels, and temperature-controlled greenhouses. These locations varied in management practices and light levels. The sampling period included a full growth cycle to analyze the changes in leaf morphology and the development of disease symptoms through the vegetative stage and the phase of fruit swelling.

**Figure 1:**
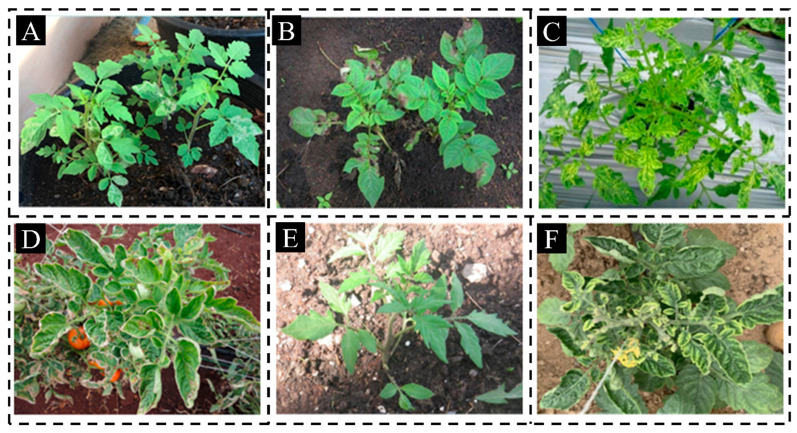
Representative images of tomato leaf diseases captured by mobile devices. (**A**) Leaf Miner; (**B**) Early Blight; (**C**) Tomato Yellow Leaf Curl Virus (TYLCV); (**D**) Late Blight; (**E**) Healthy; (**F**) Tomato Mosaic Virus. The remaining three categories (Leaf Mold, Septoria, and Spider Mites) are included in the dataset but not shown here for brevity.

Pictures were taken on an Apple iPhone 15 Pro Max. There were multiple angles and distances to capture abundant detail on the distribution of the lesion and the texture of the leaves. Pictures were taken from multiple angles and distances, including front, overhead, and side views, to capture rich detail on lesion distribution and leaf texture. To mimic the reality of a real-life farm setting, all photos were taken in ambient light, with the background left natural. The pictures were first stored in JPEG format. After collection, a manual screening procedure was conducted to eliminate blurred, overexposed, or severely occluded samples; thus, a final dataset of 2212 raw tomato leaf images was extracted. Figure 1 demonstrates representative samples.

### 2.2. Data Preprocessing

The M-TLD dataset was manually annotated primarily as a solution to the supervised training problem of the proposed detection model. LabelMe was used to complete the graphic image annotation. This was accomplished through a two-tiered system of quality control. In tier one, three volunteer undergraduate students annotated the images with bounding boxes and assigned disease categories based on a visual reference guide provided by agricultural experts. In tier two, all bounding boxes and assigned disease categories were reviewed and corrected by two agricultural experts with no fewer than five years of experience in tomato pathology. Ambiguous instances for which a disease category was indeterminate were also removed.

As LabelMe produces annotations in JSON format, they were converted to YOLO format (txt) to align with the model’s input pipeline. Each text file has a name corresponding to its image. The filtered dataset was randomly split into training, validation, and test sets in an approximately 8:1:1 proportion, resulting in 1769 training images, 221 validation images, and 222 test images. The detailed distribution of annotation instances across nine disease categories and three data splits is presented in Table 1.

As shown in Table 1, the M-TLD dataset contains 2212 images with 6581 annotation instances across 9 categories, including 8 disease types and 1 healthy class. Yellow Leaf Curl Virus has the highest number of instances (1213), reflecting its high prevalence in the sampled cultivation environments, while Late Blight has the fewest (473). The average number of annotations per image is approximately 2.97, indicating that many images contain multiple co-occurring disease instances, thereby increasing detection difficulty and better reflecting real-world field conditions. Despite the moderate class imbalance, no additional oversampling or class-weighting strategies were applied during training to maintain fairness in evaluation. The M-TLD dataset and all annotation files are publicly available at https://github.com/zhuojiaxiong6/DETR (accessed on 29 April 2026) to facilitate reproducibility and future research.

## 3. Methods and Experimental Design

### 3.1. The Overall Framework of GBR-DETR

**Figure 2:**
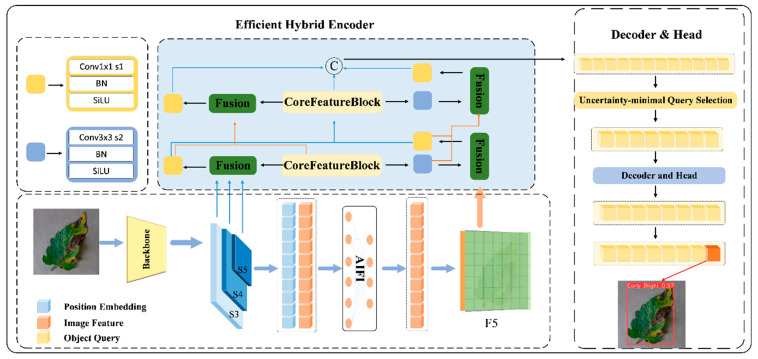
Overall architecture of the GBR-DETR network.

This paper proposes GBR-DETR (Gradient-aware Bidirectional Retentive Detection Transformer), a real-time detection framework built upon the RT-DETR end-to-end paradigm for tomato leaf disease identification. RT-DETR is adopted as the baseline owing to its hybrid CNN–Transformer encoder, which achieves a favorable speed–accuracy trade-off, and its anchor-free set prediction mechanism, which eliminates NMS post-processing, both of which are critical for low-latency edge deployment. The model integrates three synergistic innovations that correspond to its naming: (1) “G”—MGAT-Net, a gradient-aware backbone that explicitly injects Sobel-derived edge features into the semantic hierarchy to enhance boundary sensitivity for fine-grained lesions; (2) “B”—BCPN, a bidirectional context pyramid network that concurrently fuses three pyramid levels through multi-kernel depthwise convolutions, thereby bridging cross-scale semantic gaps; and (3) “R”—RFAM, a retentive feature aggregation module that replaces RepC3 with a retention-based mechanism to capture global spatial dependencies. The end-to-end processing pipeline of GBR-DETR proceeds as follows. Given an input image of size 640 × 640, the image first passes through the MGAT-Net backbone, where the Gradient Pyramid Generator (GPG) extracts multi-scale Sobel-based edge features from the stem layer and injects them into each backbone stage via the Edge-Semantic Fusion Module (ESFM). The backbone produces three hierarchical feature maps at different resolutions: P_3_ H×W, P_4_ $\frac{H}{2}\times \frac{W}{2}$, and P_5_ $\frac{H}{4}\times \frac{W}{4}$. These multi-scale features are then fed into the BCPN neck, where the CoreFeatureBlock aligns all three levels to a unified intermediate resolution, concatenates them, and applies multi-kernel depthwise convolutions to capture contextual dependencies across different receptive fields. In both the bottom-up and top-down fusion paths of the neck, RFAM replaces the original RepC3 modules with retention-based blocks to establish global spatial dependencies while preserving local textural features. Finally, the fused multi-scale features are passed to the RT-DETR decoder, which employs a Transformer-based set prediction mechanism with bipartite matching to directly output the disease category and bounding box coordinates for each detected lesion without requiring anchor design or NMS post-processing. The overall architecture is illustrated in Figure 2.

### 3.2. Bidirectional Context Pyramid Network (BCPN)

**Figure 3:**
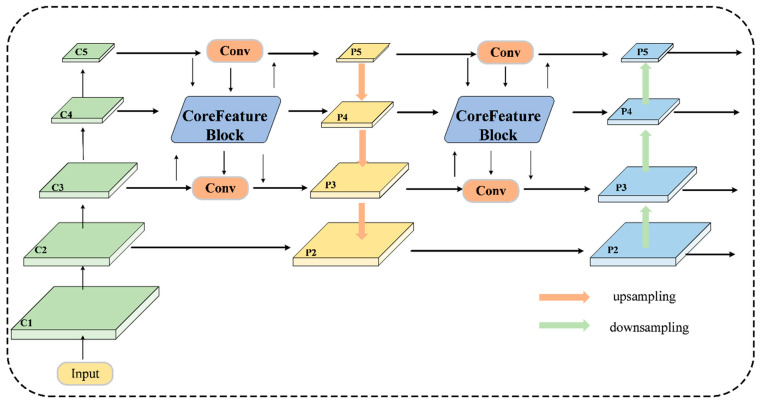
The overall workflow of the Bidirectional Context Pyramid Network (BCPN).

**Figure 4:**
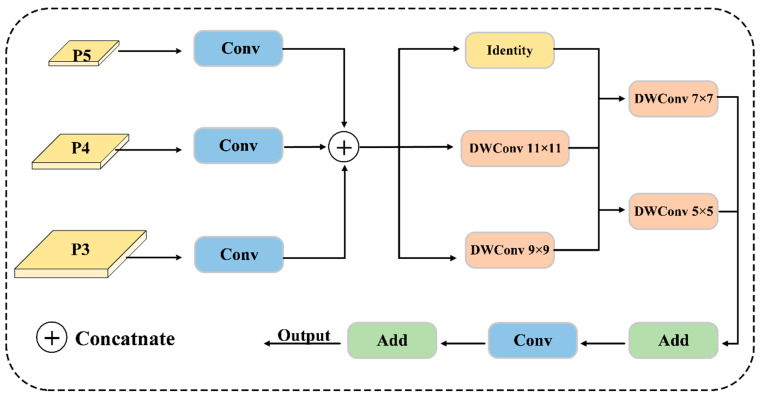
The design of the CoreFeatureBlock (CFB) module.

Traditional feature pyramid architectures, such as PANet and the Hybrid Encoder in RT-DETR, have inherent limitations when faced with fine-grained tomato leaf disease detection. The pathological appearance of tomato foliage, e.g., bacterial spots and early blight lesions, is usually characterized by small spots with subtle surface differences. The influence of the standard pyramidal structures causes information to be attenuated through sequential downsampling, thereby diluting fine-grained pathological signatures. Moreover, the sequential fusion scheme used by traditional pyramids fails to exploit multi-resolution contextual dependencies simultaneously, limiting the network’s ability to detect diseases with faint manifestations. We suggest overcoming these drawbacks by introducing the Bidirectional Context Pyramid Network (BCPN), as shown in Figure 3. whose fundamental building block, the CoreFeatureBlock, enables multi-scale interaction among features in a synergistic manner, as detailed in Figure 4.

Let $F_{P_{3}}\in R^{C_{3}\times H\times W}$,$\;F_{P_{4}}\in R^{C_{4}\times \frac{H}{2}\times \frac{W}{2}}$, and $F_{P_{5}}\in R^{C_{5}\times \frac{H}{4}\times \frac{W}{4}}$ denote input feature maps from three pyramid levels. The CoreFeatureBlock first aligns all inputs to the intermediate resolution of P_4_ ($\frac{H}{2}\times \frac{W}{2}$) through complementary spatial transformations:

(1) $$F_{P_{5}}^{\prime }={\mathrm{Conv}}_{1\times 1}({\mathrm{Upsample}}_{2\times }(F_{P_{5}}))$$

(2) $$F_{P_{4}}^{\prime }={\mathrm{Conv}}_{1\times 1}(F_{P_{4}})$$

(3) $$F_{P_{3}}^{\prime }=\mathrm{ADown}(F_{P_{3}})$$

where ${\mathrm{Upsample}}_{2\times }(\cdot )$ denotes bilinear upsampling with scale factor 2, which increases the spatial resolution of $F_{P_{5}}$ from $\frac{H}{4}\times \frac{W}{4}$ to $\frac{H}{2}\times \frac{W}{2}$, and the Advanced Downsampling (ADown) module is our proposed Advanced Downsampling module, which reduces the spatial resolution of $F_{P_{3}}$ from H×W to $\frac{H}{2}\times \frac{W}{2}$. Through these complementary operations, all three feature maps are spatially aligned to the intermediate resolution of P4 ($\frac{H}{2}\times \frac{W}{2}$), enabling subsequent channel-wise concatenation.

The ADown module synergistically integrates complementary pooling strategies to preserve heterogeneous feature characteristics. Given input $X\in R^{C_{in}\times H\times W}$, preliminary average pooling is first applied to obtain $\tilde{X}=\mathrm{AvgPool}(X)$. The smoothed feature map is then split along the channel dimension into ${\tilde{X}}_{1}$ and ${\tilde{X}}_{2}$, which then undergo strided convolution and max pooling, respectively. The complete ADown operation is formulated as:


(4) $$\mathrm{ADown}(X)=\mathrm{Concat}\left({\mathrm{Conv}}_{3\times 3}^{s=2}({\tilde{X}}_{1}),{\mathrm{Conv}}_{3\times 3}(\mathrm{MaxPool}({\tilde{X}}_{2}))\right)$$

This dual-pathway design enables ADown to capture both smoothed contextual information and salient feature responses, providing superior information retention compared to traditional downsampling.

After spatial correspondence, the three projected feature maps are concatenated as $F_{cat}=Concat(F_{P_{5}}^{\prime },F_{P_{4}}^{\prime },F_{P_{3}}^{\prime })$. To capture multi-scale contextual relations, we use a multi-kernel depthwise convolutional aggregation mechanism. Let K = {5, 7, 9, 11} represent the set of kernel sizes, covering receptive fields from local lesion textures (k = 5) to broader contextual regions (k = 11). The aggregated feature is computed as:


(5) $$F_{agg}=F_{cat}+\sum_{k\in K}\mathrm{DWCon}v_{k}(F_{cat})$$

${\mathrm{DWConv}}_{k}(\cdot )\;$ means depthwise convolution with kernel size k. The final output is obtained by a pointwise convolution followed by a residual connection:


(6) $$F_{out}=F_{cat}+\mathrm{Con}v_{1\times 1}(F_{agg})$$

The proposed BCPN presents several benefits in detecting diseases at a fine-grained level. The concurrent synthesis of elements from the three pyramid levels enables the exploitation of high-resolution spatial content in conjunction with semantically enhanced contextual content. The multi-kernel depthwise convolutions produce contextual dependencies at different levels of the receptive field, with significant variability in the lesion dimensions being fitted. Information attenuation has been addressed in the ADown module through a dual-pathway architecture, and the residual connection helps ensure stable gradient propagation during optimization.

### 3.3. Multi-Scale Gradient-Aware Transfer Network (MGAT-Net)

Conventional backbone networks used in object detection systems, including ResNet and its derivatives, consist mainly of hierarchical convolutional operations that derive semantic representations at progressively lower resolutions. Although this paradigm has proven successful in overall object detection, it has inherent disadvantages when applied to fine-grained tomato leaf disease detection. In particular, the pathological edges between diseased and healthy tissue can be considered very important discriminative features that cannot be adequately represented by simple convolutional kernels, which are pre-optimized to process texture and color patterns. The gradual gradient activity of early-stage infection, especially bacterial specks and impending fungal lesions, is gradually diffused by repeated pooling and striding, rendering the network unresponsive to diagnostically relevant edge data. Moreover, traditional backbones process features in a feed-forward manner without any mechanism to maintain and carry gradient-sensitive representations across different scales. To overcome these shortcomings, we introduce the Multi-Scale Gradient-Aware Transfer Network (MGAT-Net). This more powerful backbone architecture explicitly leverages edge information as it ascends the feature hierarchy. The innovation comprises two complementary modules: the Gradient Pyramid Generator (GPG) and the Edge-Semantic Fusion Module (ESFM).

The Gradient Pyramid Generator obtains multi-scale edge representations that capture pathological boundaries at different spatial granularities. Given the input feature map $F_{in}\in R^{C\times H\times W}$ from the stem layers, GPG applies standard 3 × 3 Sobel operators in the horizontal $G_{x}$ and vertical $G_{y}$ directions to extract gradient information. Compared with learnable edge detectors, Sobel operators provide deterministic, parameter-free gradient extraction, avoiding additional training overhead and ensuring stable boundary responses across varying lesion morphologies. The gradient response is computed through depthwise 2D convolution, where each channel is convolved independently with fixed Sobel kernels to preserve channel-wise independence:


(7) $$E={\mathrm{DWConv}}_{G_{x}}(F_{in})+{\mathrm{DWConv}}_{G_{y}}(F_{in})$$

$E\in R^{C\times H\times W}$ denotes the encoded gradient information of the edge-enhanced feature map across all channels. This step is followed by GPG, which generates a multi-scale edge pyramid through successive max-pooling operations. In a pyramid where levels have N features $\left\{P_{3},{\;P}_{4},\;P_{5}\right\}$, the edge features of each scale are computed as:


(8) $$E^{\left(i\right)}={\mathrm{MaxPool}}_{2\times 2}\left(E^{\left(i-1\right)}\right),i=1,\ldots ,N,\;\mathrm{where}\;E^{\left(0\right)}=E$$

Each level of the pyramid is then projected to the corresponding channel dimension using 1×1 convolution:

(9) $${\hat{E}}^{\left(i\right)}={\mathrm{Conv}}_{1\times 1}^{C_{i}}\left(E^{\left(i\right)}\right),\;{\hat{E}}^{\left(i\right)}\in R^{C_{i}\times H_{i}\times W_{i}}$$

where $C_{i}$ represents the channel dimension at the i-th pyramid level, and this channel dimension is associated with the i-th stage of the backbone.

The Edge-Semantic Fusion Module combines gradient features with semantic features produced by the backbone stages. At every scale, ESFM receives two inputs: the edge feature $\hat{E}(i)$ of GPG and the semantic feature S(i) of the previous block of the backbone. The fusion procedure concatenates the edge feature ${\hat{E}}^{\left(i\right)}$ and semantic feature $S^{\left(i\right)}$, then performs sequential channel compression (1 × 1 conv), local pattern extraction (3 × 3 conv), and channel expansion (1 × 1 conv). The complete ESFM operation is formulated as:


(10) $$\mathrm{ESFM}({\hat{E}}^{\left(i\right)},S^{\left(i\right)})={\mathrm{Conv}}_{1\times 1}({\mathrm{Conv}}_{3\times 3}({\mathrm{Conv}}_{1\times 1}(\mathrm{Concat}({\hat{E}}^{\left(i\right)},S^{\left(i\right)}))))$$

**Figure 5:**
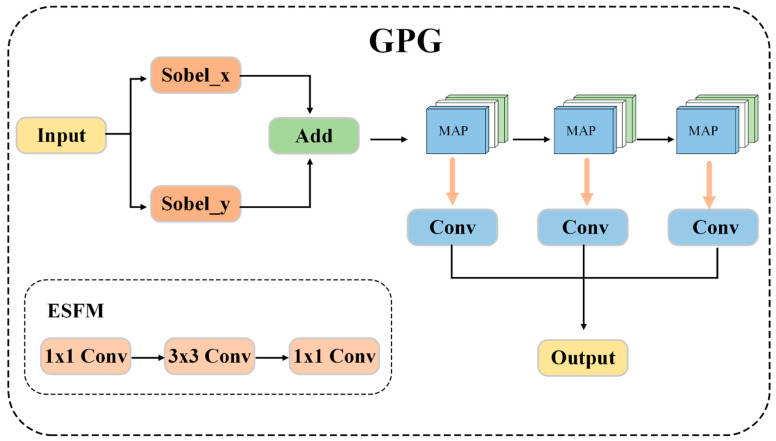
The architecture of the MGAT-Net module.

The proposed MGAT-Net architecture offers several unique benefits for fine-grained disease detection. First, explicitly accentuating gradient details with Sobel convolution helps ensure that fine pathological boundaries are retained and propagated across the network hierarchy, thereby alleviating the edge-loss characteristic of traditional backbones. Second, the pyramid of multi-scale edges enables scale-invariant representation of boundaries and accommodates large differences in lesion sizes observed across diverse disease categories and at different stages of progression. Third, combining edge and semantic representations at every backbone phase enables fusion of complementary information: gradient-sensitive representations enhance localization accuracy, whereas semantic features add categorical discrimination. Fourth, both GPG and ESFM are designed to be lightweight, introducing minimal computational overhead while significantly enhancing the network’s sensitivity to fine-grained pathological manifestations. The specific architecture of the MGAT-Net module is shown in Figure 5.

### 3.4. Retentive Feature Aggregation Module (RFAM)

**Figure 6:**
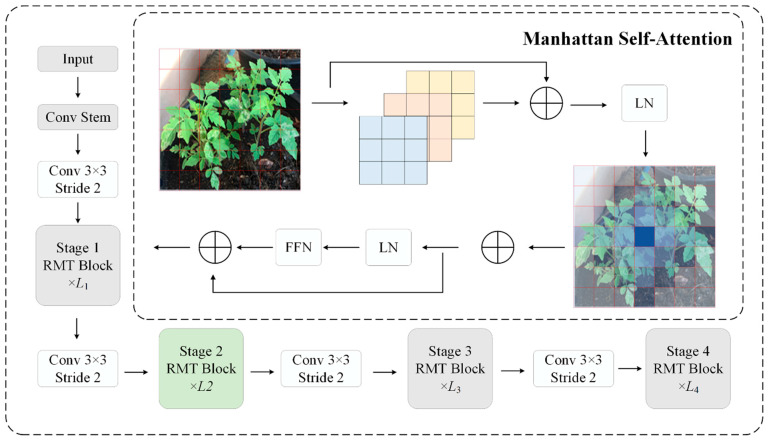
Architecture diagram of RFAM.

The RepC3 module, commonly used in RT-DETR for feature aggregation in the feature pyramid network, uses reparameterized convolution to learn local patterns from concatenated multi-scale features. Although this design achieves computational efficiency via structural reparameterization at inference time, it has inherent shortcomings in manipulating fine-grained disease features. In particular, the network’s entirely convolutional structure is limited in that it restricts the receptive field to local regions of a larger image and excludes capturing long-range spatial interactions among those regions, thereby hindering its ability to distinguish between visually similar disease categories. Generally, pathological patterns on tomato leaves appear as spatially localized lesions with typical patterns, e.g., the concentric ring patterns of early blight or the random distribution of bacterial specks, which require global contextual reasoning and are not available to local convolutions alone. In addition, the lack of explicit positional encoding in RepC3 makes the module insensitive to the spatial organization of pathological features, which is an essential discriminative cue for fine-grained disease classification. To address these shortcomings, we introduce the Retentive Feature Aggregation Module (RFAM), as illustrated in Figure 6, which uses the Retentive Block (RetBlock) architecture proposed in RMT [24] as part of the C3 architecture, enabling efficient long-range dependency modeling with linear computational complexity.

The retention mechanism, originally introduced in RetNet [25] for language modeling and later adapted to vision tasks by RMT [24], provides an efficient alternative to standard self-attention. It employs multi-head chunkwise recurrent computation with a learnable decay factor and RelPos2d relative positional encoding, achieving global receptive-field coverage at linear complexity O(HW) rather than the quadratic O(H^2^W^2^) of vanilla self-attention. We refer readers to Fan et al. [24] for the detailed formulation.

Given the input feature map $F\in R^{C\times H\times W}$, the retention output is combined with a parallel convolutional branch via residual addition:

(11) $$F_{out}={\mathrm{Conv}}_{1\times 1}\left(F_{ret}+{\mathrm{Conv}}_{1\times 1}(F)\right)$$

where $F_{ret}$ denotes the output of the retention operation. This dual-branch structure enables the simultaneous capture of global contextual patterns (via retention) and local textural features (via convolution).

Adding RFAM to the feature pyramid network replaces all RepC3 modules in both the bottom-up and top-down directions, enabling each level of fusion to capture global contextual aggregation. Several benefits arise from this architectural change for fine-grained disease detection. First, the retention mechanism can store long-range spatial dependencies with a linear complexity of O(HW) compared to the quadratic complexity of $O(H^{2}W^{2})$ of simple self-attention. Second, the explicit relative positional encoding by RelPos2d retains spatial configuration data essential for differentiating disease patterns. Third, the chunkwise recurrent formulation enables efficient processing and global receptive field coverage. Fourth, the dual-branch architecture, combining retention and convolution, enables the extraction of both global contextual patterns and local textural features.

### 3.5. Configuration of the Experimental Environment

All training and optimization processes for the object detection model were conducted on a high-performance workstation running Ubuntu 22.04 LTS. The hardware and software configuration of the experimental environment is shown in Table 2.

### 3.6. Evaluation Indicators

We adopt standard object detection evaluation metrics in this study. Precision (P) and Recall (R) measure the accuracy and completeness of positive predictions, respectively, and the F1-score represents their harmonic mean. Average Precision (AP) is computed as the area under the Precision–Recall curve for each class, and mean Average Precision (mAP) is the average of AP values across all classes. In this work, we report mAP at IoU thresholds of 0.50 (mAP_50_) and 0.75 (mAP_75_), as well as mAP_50–95_, averaged over IoU thresholds from 0.50 to 0.95 in increments of 0.05, following the Ultralytics evaluation protocol. Inference efficiency is measured by Frames Per Second (FPS) and average per-image latency in milliseconds.

## 4. Results

### 4.1. Implementation Details

All images were resized to 640 × 640 before model training to ensure uniform input data. In addition to offline augmentation, online augmentation was used during model training to ensure that real-time transformations were applied. This increased the diversity of the data and the model’s ability to generalize. The training hyperparameters (Table 3) followed the default RT-DETR configuration with minor adjustments: SGD with linear learning rate decay from 0.01 to 0.0001 was adopted for stable convergence on the relatively small-scale M-TLD dataset; the batch size of 16 was determined by GPU memory constraints (24 GB); and early stopping with patience of 15 epochs was employed to prevent overfitting. The input resolution of 640 × 640 was selected to balance detection accuracy and edge inference speed, as further validated in Section 4.4. To ensure statistical reliability, all experiments were conducted five times independently using different random seeds (42, 123, 456, 789, 1024). All results are shown as the average of the five experiments.

### 4.2. Ablation Study

To validate the GBR-DETR design thoroughly, a three-step ablation study was conducted. The first of the three levels focused on submodule structure. At this level, a hyperparameter of the BCPN submodule CFB—a critical component optimally set by configuring the rectangular hyperparameter kernel size—was optimized. The next level focused on the integration of BCPN, MGAT-Net, and RFAM into the baseline RT-DETR. At this level, the contribution of each module and the cumulative effect on the baseline model were quantified. The last level was the qualitative analysis. Grad-CAM heatmaps were used to present evidence evaluating the proposed model and its techniques in the attention to the shaping of lesion regions.

The diversity of receptive fields for multi-scale lesion perception was defined by the flexible kernel configuration of CFB, as shown in Table 4. Using a single kernel (5) yielded the worst mAP_50_ of 67.85%, as a fixed 5 × 5 receptive field could only respond to lesions within a small range of sizes and did not capture small spots or large necrotic patches. Introducing a 7 × 7 branch (5, 7) lifted mAP_50_ to 75.09%, since the two complementary scales jointly covered the dominant lesion-size distribution and produced more discriminative fused features. Counterintuitively, further adding a 9 × 9 kernel (5, 7, 9) caused a slight drop to 72.97%; we attributed this to the heavy overlap between adjacent receptive fields—when three closely spaced kernels responded to similar regions, the fused representation became redundant rather than complementary, weakening gradient signals during training. The drawback was reduced by extending the receptive-field spectrum to large coalesced lesions and restoring scale complementarity using the 11 × 11 kernel (5, 7, 9, 11), achieving the best mAP_50_ of 75.81%. However, extending the kernel size to (5, 7, 9, 11, 13) decreased mAP_50_ to 68.43% at the cost of greater GFLOPs. The mAP_50_ decreased because the 13 × 13 kernel exceeds the typical lesion size in our data and acts as a low-pass filter, smoothing out high-frequency components such as spot edges and chlorotic halos that are critical for discriminating diseases. Thus, the configuration (5, 7, 9, 11) was adopted as the default CFB setting, which provided the optimal trade-off between scale complementarity and feature sharpness.

With the optimal CFB configuration established above, we further evaluated the overall effectiveness of BCPN, MGAT-Net, and RFAM by progressively integrating them into the baseline. The results are summarized in Table 5.

To confirm the effectiveness of the proposed BCPN module, the baseline RT-DETR model achieved an mAP_50_ of 74.03%, a precision of 75.28%, and a recall of 72.25%, with 19.88 M parameters and 57 GFLOPs. With the addition of the BCPN module, mAP_50_ rose to 75.81%, and precision and recall also rose to 79.58% and 73.23%, respectively, while mAP_75_ and mAP_50–95_ were 65.39% and 60.80%. Despite increasing the number of parameters by 2.36 M (11.88%) and computational cost by 9.1 GFLOPs (15.96%), the results of the BCPN module were significantly better. Building on the BCPN baseline model, the MGAT-Net module further optimized boundary refinement. Parameters and GFLOPs increased to 23.48 M and 68.6, respectively, whereas mAP_50_ and mAP_75_ went to 77.15% and 68.07%, respectively. Lastly, this was the most balanced performance that the introduction of RFAM achieved. The precision increased to 82.14%, the highest among all configurations, and the F1-score improved to 76.93%. Simultaneously, the number of parameters dropped to 22.13 M (1.35 M fewer than BCPN+MGAT-Net), and GFLOPs dropped to 59.4 (an increase of 2.4 GFLOPs over the baseline).

In addition to the quantitative assessment in Table 5, we generated feature activation heatmaps using Grad-CAM to further visualize the internal mechanisms and effectiveness of the proposed BCPN, MGAT-Net, and RFAM modules.

**Figure 7:**
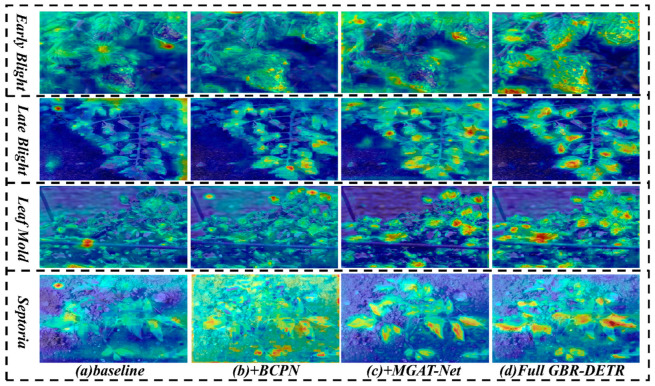
Grad-CAM activation heatmaps illustrating the progressive effect of each proposed module. From left to right: baseline RT-DETR, +BCPN, +BCPN+MGAT-Net, and the complete GBR-DETR. Warmer colors (red/yellow) indicate regions of higher model attention, while cooler colors (blue) indicate regions of lower attention.

As shown in Figure 7, the integration of additional proposed modules yielded a clear progression in how the model concentrated its attention. In the baseline model, we observed that the model’s attention was dispersed and interfered with by the background. As an improvement, we observed the integration of MGAT-Net producing an increase in the intensity of the model’s attention around the edges of the lesion. The design of MGAT-Net was targeted at edge-aware feature transfer in the model. In addition, RFAM minimized the background noise around the model in the GBR-DETR framework by establishing global spatial dependencies. These enhancements produced a visual focusing improvement toward the edges of the lesion, hence supporting the conclusion that the cornerstone design of the GBR-DETR framework is the focusing capability toward diagnostically relevant pathological components.

### 4.3. Comparison with Other Classic Object Detection Models

To provide a comprehensive analysis of the detection accuracy and real-time performance of GBR-DETR, we systematically compared it with 13 popular object detection models trained on the M-TLD dataset. The chosen baselines covered a broad variety of architectures: the classic two-stage detector Faster-RCNN [26], and sophisticated single-stage or anchor-free systems like ATSS [27], GFL [28], TOOD [29], and VFNet [30]. We also included the transformer-based DINO [31], the lightweight YOLOX-tiny [32], as well as the latest versions of the YOLO family, namely, YOLOv8m [33], YOLOv9m [34], YOLOv10m [35], YOLOv11m [36], YOLOv26m [37], and Hyper-YOLO [38]. Each model was evaluated under comparable experimental conditions, without using any pre-trained weights, to ensure a fair comparison of their detection accuracy and computational efficiency. The comparison results are presented in Table 6.

GBR-DETR achieved the best overall results compared to the other frameworks in this area. The model had 22.13 M parameters with an mAP_50_ of 78.91%, mAP_75_ of 68.52%, mAP_50–95_ of 62.98%, 82.14% precision, a 72.91% recall, and an F1-score of 76.93% and 59.4 G FLOPs.

Among single-stage detectors, ATSS and GFL achieved mAP_50_ of only 46.8% and 48.2%, respectively, indicating limited capability in capturing fine-grained disease features. TOOD achieved a recall of 62.3%, but its precision of only 53.1% led to excessive false positives. VFNet attained an mAP_50_ of 68.4% with relatively balanced precision and recall, yet remained substantially inferior to GBR-DETR. The transformer-based DINO model required 47.557 M parameters and 193 G FLOPs but achieved an mAP_50_ of only 52.9%, suggesting that vanilla Transformer architectures are inefficient for plant disease detection. The two-stage detector Faster-RCNN achieved an mAP_50_ of 78.5%, but its computational cost (146 G) was 2.46 times that of GBR-DETR, making it unsuitable for edge deployment.

Within the YOLO family, YOLOX-Tiny’s computational cost was the least (7.6 G), but its detection performance was low, with an mAP_50_ of only 74.5%. YOLOv8m achieved a slightly higher mAP_75_ (68.57%) than GBR-DETR, but its lower Recall (69.38%) resulted in a higher missed-detection rate. YOLOv9m and YOLOv10m achieved 75.05% and 77.18% mAP_50_ values, respectively. Two recently proposed models were also evaluated. YOLOv26m, the latest NMS-free end-to-end YOLO variant released in 2026, achieved an mAP_50_ of only 74.98% on the M-TLD dataset despite its competitive performance on general benchmarks, indicating that its edge-optimized design may sacrifice fine-grained disease feature extraction. Hyper-YOLO, which introduced hypergraph-based feature aggregation, achieved an mAP_50_ of 75.35% with the lowest computational cost among medium-scale models (33.8 G), yet its accuracy remained 3.56 percentage points below GBR-DETR. YOLOv11m achieved the second-highest mAP_50_ of 78.51% among all compared methods. However, GBR-DETR still surpassed YOLOv11m by 0.40, 2.30, and 5.27 percentage points in mAP_50_, F1-score, and Precision, respectively, while maintaining a lower computational cost (59.4 G vs. 67.7 G).

**Figure 8:**
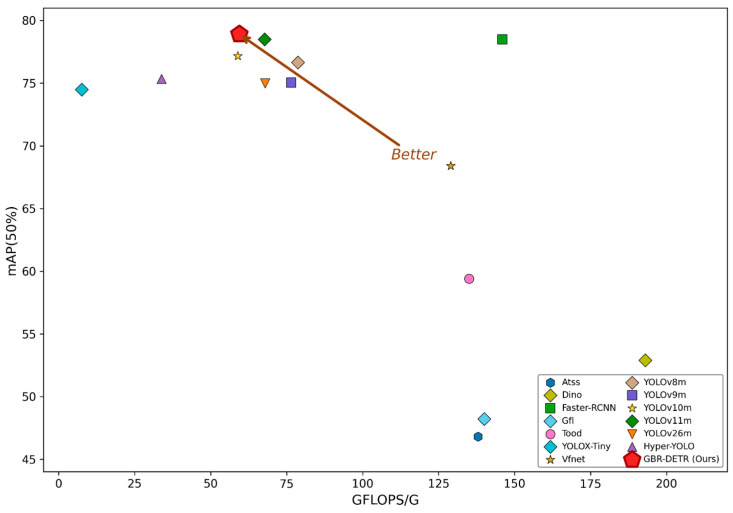
Comparison of mAP_50_ versus computational cost (GFLOPs) among different object detection models. The arrow indicates the direction of better performance (higher accuracy with lower computational cost).

**Figure 9:**
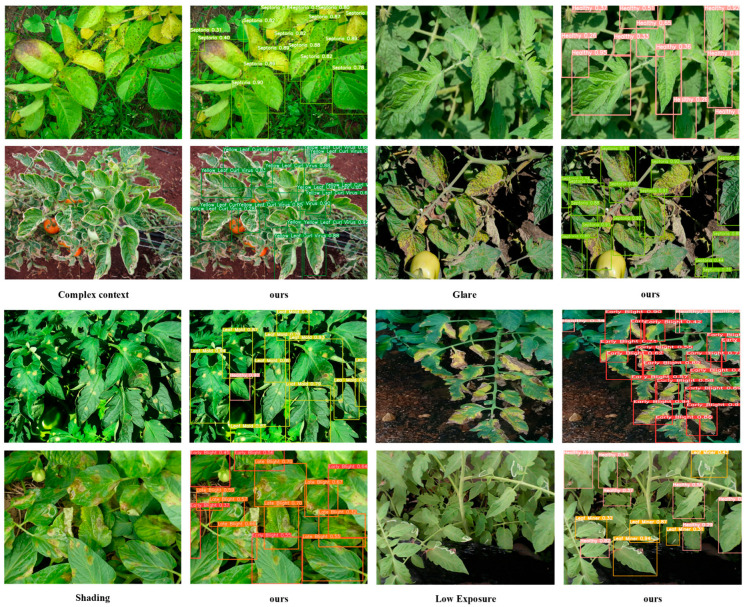
Detection results of GBR-DETR under different challenging conditions, including complex backgrounds, varying illumination, partial occlusion, and dense leaf coverage.

**Figure 10:**
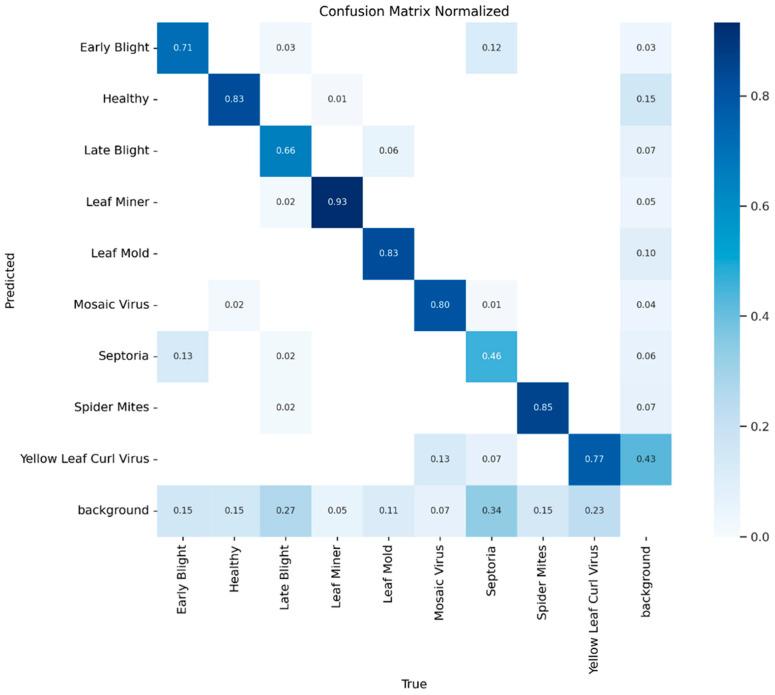
Normalized confusion matrix of GBR-DETR on the M-TLD test set. Diagonal values represent correct classification rates for each category.

Figure 8 displays a scatterplot of mAP_50_ and GFLOPs to illustrate the relationship between accuracy and efficiency. Situated in the upper-left region of the Pareto frontier, GBR-DETR achieved the best compromise between detection accuracy and computational efficiency. This made it ideal for real-time disease detection on edge devices in smart agricultural systems. GBR-DETR showed good performance even under difficult conditions such as target exposure, background clutter, and low-light occlusions, as shown in Figure 9. Figure 10 presents the normalized confusion matrix of the model on the M-TLD test set, illustrating its per-class recognition performance, with diagonal values indicating the correct classification rates for each category.

**Figure 11:**
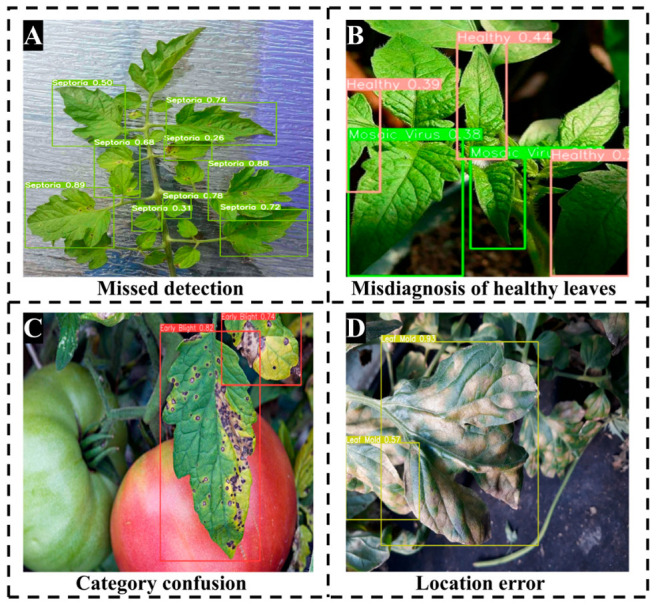
Representative failure cases of GBR-DETR on the M-TLD test set. (**A**) Missed detection of small disease spots under low foreground–background contrast. (**B**) Misdiagnosis of healthy leaves as mosaic virus due to shadow patterns on overlapping leaves. (**C**) Category confusion between late blight and early blight at advanced stages. (**D**) Positioning error caused by merging two adjacent infected leaves into one bounding box.

Figure 11 presents four typical failure cases of GBR-DETR on the M-TLD test set, revealing several limitations of the model. (A) Missed detection. In cases where the contrast between the foreground and background of the leaf was low and the background texture of the leaf was dense, the model failed to detect certain disease spots. Although MGAT-Net used the Sobel gradient prior to enhance the contrast of the boundaries, due to the dense overlapping of the leaves, a large number of chaotic texture features were generated, making it difficult for the model to recognize these small disease spots. (B) Misdiagnosis of healthy leaves. For images containing only healthy leaves, the model occasionally gave a high confidence prediction for mosaic virus. This misclassification occurred because the shadow and highlight patterns produced by the overlapping of adjacent leaves are visually similar to the mosaic virus-infected bumpy texture. This phenomenon further indicated that when the dominant gradient in the image originated from changes in lighting rather than pathological features, the high sensitivity of MGAT-Net to gradient signals may have had an adverse effect. (C) Category confusion. This case showed that the model had difficulty distinguishing late blight and early blight at the disease’s advanced stage. In the later stages of disease development, the lesion morphology, color, and spatial distribution of these two diseases are extremely similar, both mainly presenting necrotic phenomena. Although the model effectively captured long-range dependencies, its visual representation’s ability to distinguish still needed further improvement. (D) Positioning error. When two adjacent leaves were simultaneously infected with leaf blight, the spatial adhesion between the leaves caused the model to merge two independent instances into an expanded bounding box. Although the predicted category was correct, the instance quantity was inaccurate, and the bounding box became abnormal. This represented an inherent limitation of the RT-DETR model, which relied on set prediction and Hungarian matching: the model had difficulty treating visually similar and spatially adjacent instances as independent targets, and this problem was particularly evident in densely planted crops.

### 4.4. Deploying the GBR-DETR Model on NVIDIA Jetson Orin Nano

To demonstrate the feasibility of deploying the proposed model on edge devices, this paper adopted the NVIDIA Jetson Orin Nano as the inference platform. The operating environment was based on Ubuntu 22.04 and JetPack 6.2.1. The platform integrated NVIDIA’s acceleration libraries, including CUDA, cuDNN, and TensorRT, which substantially improved real-time inference efficiency in resource-constrained settings. The environment was built in Python 3.10, and dependencies for inference and visualization were also installed. Details on specific hardware and software environments can be found in Table 7.

The model deployment was a two-step process, which included ONNX export and conversion to a TRT engine. The trained PyTorch model was first exported to an intermediate ONNX (Open Neural Network Exchange) format using the Ultralytics framework on the training server. ONNX is an exchange format for deep learning models, making models cross-platform compatible. During the export process, the study created six ONNX models with varying input resolutions (320 × 320, 480 × 480, 512 × 512, 640 × 640, 800 × 800, and 1024 × 1024) based on the application scenario to enable later comparison of multi-scale performance. The ONNX models were then converted into much faster inference engines on Jetson Orin Nano devices using the TensorRT trtexec tool. It used an FP16 (half-precision floating-point) format of conversion. This reduced the bit depth of model parameters and intermediate activation values from 32-bit to 16-bit by a factor of two, without significantly compromising detection accuracy. This resulted in a 50 percent reduction in model size and a substantial reduction in the model’s memory bandwidth requirements. The optimal parameter workspace size was set to 4096 MB, which provided sufficient buffer space for computations in complex layers. Convolutions, batch normalizations, and activation functions could be automatically fused into a single operation by TensorRT; independent computation branches could be parallelized to reduce cost via horizontal layer fusion; kernel auto-tuning could be used to select the best compute cores based on the Ampere architecture GPU hardware features; the quantization range could be dynamically adjusted to minimize accuracy loss. The engine file was heavily optimized for the target hardware, thereby improving model inference performance.

To systematically analyze the trade-off between the detection accuracy and the inference speed of varying input resolutions, the present study examined the six-scale TensorRT models on the test data (222 images). The evaluation metrics were detection precision, recall, and F1-score.

**Figure 12:**
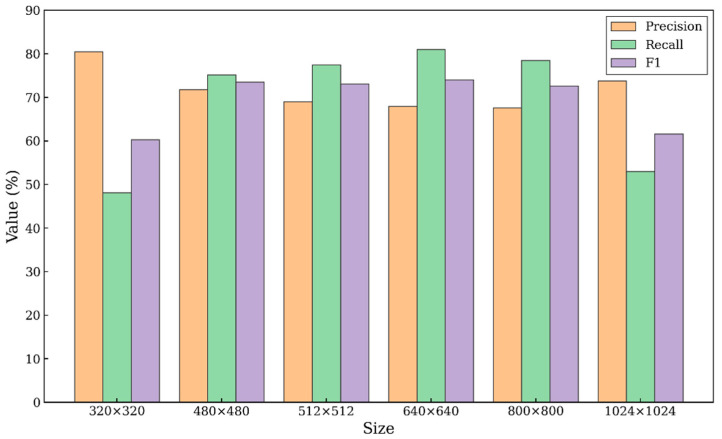
Variation in GBR-DETR accuracy metrics across different image input sizes on edge devices.

Figure 12 provides more detailed performance measurements for the multi-scale model. Regarding detection accuracy, the model’s performance first increased, peaked at 640 × 640, and slightly declined at higher resolutions due to the relatively low proportion of small objects. With such limited input information, the 320 × 320 model achieved only an F1-score of 0.6033, whereas the 640 × 640 model reached 0.7395, which was well-balanced in terms of Precision and Recall. There were diminishing marginal returns to further increasing the resolution to 1024 × 1024 to achieve increased accuracy. The overall trend in object detection experiments could explain this. At very high resolutions, more detailed information could be detected, but the benefits diminished in datasets where the proportion of small objects was relatively low.

**Figure 13:**
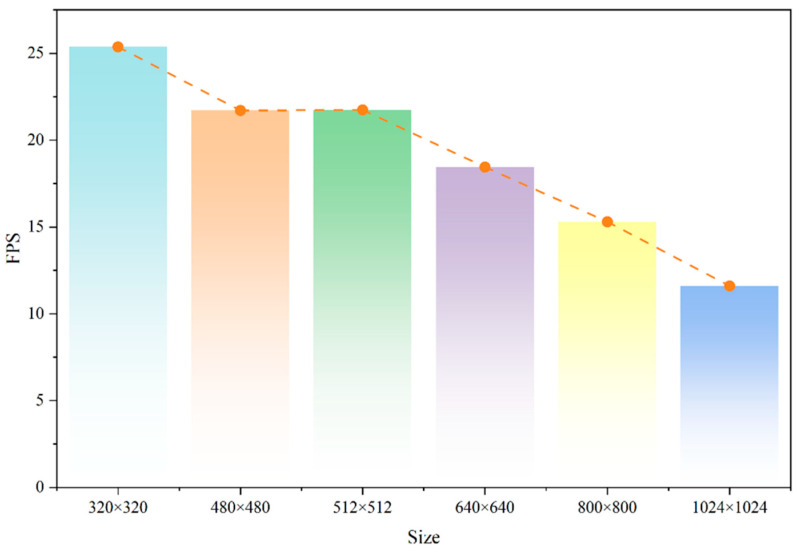
Speed differences of GBR-DETR on edge devices with various image input sizes.

**Figure 14:**
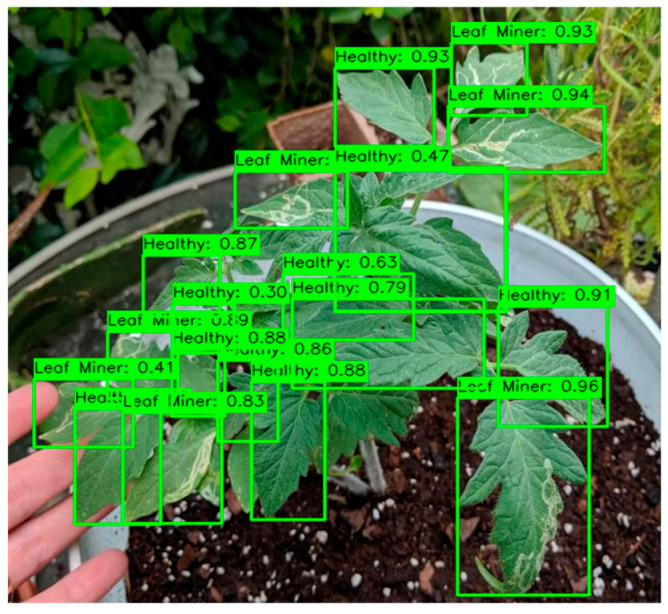
GBR-DETR’s actual detection results on edge devices.

Figure 13 confirms that the model’s inference speed (FPS) decreased significantly as the input image size increased. This was mainly because higher-resolution images have more pixel features to process, making the computation very demanding and thus reducing inference speed. As an example, when the resolution was increased from 320 × 320 to 1024 × 1024, FPS was reduced by more than twofold to 11.60, which was about half, demonstrating that the model could not perform real-time inference at high resolutions. This made it difficult to use resource-constrained edge devices. Hence, as a trade-off between detection and inference speed, this paper finally settled on 640 × 640 as the default image size for the model. This aspect guaranteed a high detection rate and tolerably steady and satisfactory real-time inference rates. The actual detection performance of GBR-DETR deployed on the edge device is further illustrated in Figure 14.

## 5. Discussion

### 5.1. Architectural Advantages and Mechanism Analysis

The proposed GBR-DETR framework is superior at detecting tomato leaf diseases, primarily due to synergistic architectural innovations that address the drawbacks of conventional CNN-based detectors. Single-stage detectors such as YOLOv11 are faster at making predictions but are often strongly affected by semantic–spatial misalignment in complex backgrounds, leading to the loss of fine-grained details. GBR-DETR addresses this through the Bidirectional Context Pyramid Network (BCPN), which creates a recursive network of information highways. Through a strong association between high-level semantics and low-level texture, BCPN ensures that small, early lesions survive the feature downsampling process.

Moreover, the use of the Retentive Feature Aggregation Module (RFAM), in contrast to two-stage detectors (e.g., Faster R-CNN), facilitates global modeling in GBR-DETR, since such models are typically built on local convolution operations. This capability is particularly important in tomato fields, where dense foliage and occlusion are common. The Manhattan distance spatial retention mechanism preserves semantic coherence and provides long-range contextual modeling, allowing the model to distinguish neighboring lesions across overlapping leaves—a task that pure CNN architectures typically struggle with. Meanwhile, the direct use of the end-to-end RT-DETR paradigm eliminates the need for heuristic anchor design or NMS post-processing. This simplification eases the inference pipeline and has a minimal effect on latency jitter, making the prediction time of the model much more predictable for integration into a robotic intervention system.

### 5.2. Generalization and Cross-Domain Robustness

To test how the model can be applied to a domain different from that of the M-TLD dataset, we conducted a cross-domain test on the publicly available PlantDoc dataset [39]. PlantDoc images are web-scraped, unlike our high-quality, in-house-collected samples, and are low-resolution, with compression artifacts and high background diversity. This presents a major challenge arising from domain gaps. The cross-domain performance of GBR-DETR and RT-DETR on the PlantDoc dataset is summarized in Table 8.

As shown, GBR-DETR performs remarkably well in this difficult task. It achieved a Precision of 61.61% and mAP_50_ of 48.60%, substantially higher than the RT-DETR baseline (Precision: 60.01%, mAP_50_: 46.49%). This improvement is important; it indicates that the MGAT-Net module stops the model from being too specific to the source domain’s color or texture distribution. Instead, the model learns general structural codes of lesions (e.g., the rings of Early Blight) by explicitly encoding gradient boundaries and is not dataset-dependent. This emphasis on structural features enables GBR-DETR to adapt to new images and potentially to other solanaceous plants (with minor fine-tuning), and hence the need for it in practical applications.

### 5.3. Limitations and Future Prospects

Despite the promising findings, several limitations remain and warrant further investigation to broaden the model’s applicability. First, regarding environmental diversity, the available data do not include samples under extreme weather conditions, i.e., heavy fog, condensation on leaves, or sudden variations in lighting intensity during storms. These circumstances can affect the effectiveness of MGAT-Net gradient extraction and may require additional data augmentation or domain adaptation models. Second, regarding computational limitations, the model can operate in real time (around 18.45 FPS) on a single Jetson Orin Nano; however, it is difficult to scale to large greenhouses and to use multiple cameras to capture concurrent streams. The computational workload (59.4 GFLOPs) is much more demanding than that of a relatively small model such as YOLOX-Tiny and may not be compatible with low-end microcontrollers. Third, the model is not well interpreted. Although attention maps provide insight into areas of interest, they do not explain the disease condition in biological terms, which is very important for gaining the trust of agronomists and farmers.

Further efforts in future work will focus on the following directions: (1) developing multi-modal data models to add depth (RGB-D) and multispectral data to make the models more robust to illumination variability; (2) research into model compression methods (e.g., knowledge distillation and channel pruning) to further simplify edge cluster predictions; and (3) the addition of Explainable AI (XAI) modules to visualize pathological features and bridge the gap between deep learning predictions and pathological diagnosis.

## 6. Conclusions

This paper focuses on the current problem of reliable detection of tomato leaf diseases in a complex farm environment. Here, a real-time detection model for tomato leaf disease identification, GBR-DETR (Gradient-aware Bidirectional Retentive Detection Transformer), is introduced. It is based on the RT-DETR end-to-end architecture, but incorporates three complementary structural design innovations to address the so-called semantic–spatial misalignment and local receptive field issues of current detectors.

Specifically, the Bidirectional Context Pyramid Network (BCPN) recursively establishes an information path between high-level semantics and low-level textures, thus improving fine-grained lesion recognition. The Multi-Scale Gradient-Aware Transfer Network (MGAT-Net) directly fuses gradient priors (using Sobel operators), facilitating accurate boundary detection under various lighting conditions. Additionally, the Retentive Feature Aggregation Module (RFAM) uses a spatial decay function to model global information, helping to separate nearby lesions in crowded and cluttered environments.

The experimental findings from the self-built M-TLD dataset reveal that GBR-DETR achieves a Precision of 82.14%, a Recall of 72.91%, and an F1-Score of 76.93%, which are higher than those of state-of-the-art baselines, including YOLOv11m and Faster-RCNN. The PlantDoc dataset further demonstrates the model’s high capacity to generalize (mAP_50_: 48.6%) in cross-domain evaluation. Importantly, deployment experiments on the NVIDIA Jetson Orin Nano demonstrate the framework’s engineering feasibility: with FP16 acceleration via TensorRT, the model can run at a constant inference rate of 18.45 FPS (latency of about 54 ms) at 640 × 640 resolution.

Overall, GBR-DETR represents a favorable choice for achieving the desired balance among detection accuracy, robustness, and computational efficiency, and is a viable option for intelligent disease-monitoring systems within the greenhouse. The next stage of research is to expand the dataset by means of multi-modal data (RGB-D) and to research lightweight model compression methods for deployment on ultra-low-power edge computers.

## Figures and Tables

**Table 1:** Distribution of annotation instances per disease category in the M-TLD dataset.

Category	Train	Val	Test	Total
Early Blight	646	52	84	782
Healthy	614	47	75	736
Late Blight	371	64	38	473
Leaf Miner	675	75	86	836
Leaf Mold	635	53	103	791
Mosaic Virus	504	76	74	654
Septoria	447	89	62	598
Spider Mites	431	55	12	498
Yellow Leaf Curl Virus	980	128	105	1213
Total	5303	639	639	6581
Images	1769	221	222	2212

**Table 2:** Experimental environment hardware and software configuration.

Name	Configuration Information
operating system	Ubuntu 22.04 (Canonical Ltd., London, UK)
Development language	Python 3.10 (Python Software Foundation, Wilmington, DE, USA)
Framework	Pytorch 1.13.1 (Meta AI, Menlo Park, CA, USA)
CPU	Intel (R) Xeon(R) Platinum8352V (Intel Corporation, Santa Clara, CA, USA)
GPU	NVIDIA GeForce RTX 4090 (24 GB) (NVIDIA Corporation, Santa Clara, CA, USA)
memory	120 GB (Samsung Electronics Co., Ltd., Suwon, Republic of Korea)

**Table 3:** Details of the final model parameter settings.

Parameter	Setting
Initial Learning Rate	0.01
LR Scheduler	Linear lr
Final Learning Rate	0.0001
Optimizer	SGD
Workers	4
Batch size	16
Epoch	300
Patience	15

**Table 4:** The impact of different kernel sizes on the performance of CFB.

Kernel Sizes	Precision	Recall	F1	mAP_50_	mAP_75_	mAP_50–95_	Parameters (M)	GFLOPs (G)
(5)	0.7414	0.6565	0.6904	0.6785	0.5623	0.5152	22	65.3
(5, 7)	0.7871	0.7278	0.7534	0.7509	0.6503	0.6085	22.05	65.5
(5, 7, 9)	0.821	0.6793	0.7377	0.7297	0.6224	0.5773	22.13	65.7
(5, 7, 9, 11)	0.7958	0.7323	0.7602	0.7581	0.6539	0.608	22.24	66.1
(5, 7, 9, 11, 13)	0.7325	0.6645	0.6919	0.6843	0.5553	0.5194	22.41	66.6

**Table 5:** Results of the ablation experiment.

BCPN	MGAT-Net	RFAM	P	R	F1	mAP_50_	mAP_75_	mAP_50–95_	Parameters (M)	GFLOPs (G)
×	×	×	0.7528	0.7225	0.7319	0.7403	0.6511	0.5986	19.88	57
√	×	×	0.7958	0.7323	0.7602	0.7581	0.6539	0.608	22.24	66.1
√	√	×	0.7786	0.7373	0.756	0.7715	0.6807	0.6235	23.48	68.6
√	√	√	0.8214	0.7291	0.7693	0.7891	0.6852	0.6298	22.13	59.4

Note: “√” indicates that the corresponding module is included in the model, while “×” indicates that it is not.

**Table 6:** Comparison of the performance of GBR-DETR with that of other models.

Method	Param (M)	FLOPs (G)	P	R	F1	mAP_50_	mAP_75_	mAP_50–95_
Atss	32.132	138	0.413	0.533	0.438	0.468	0.397	0.355
Dino	47.557	193	0.511	0.528	0.505	0.529	0.46	0.423
Faster-RCNN	41.389	146	0.736	0.711	0.717	0.785	0.643	0.559
Gfl	32.277	140	0.465	0.501	0.441	0.482	0.413	0.372
Tood	32.037	135	0.531	0.623	0.558	0.594	0.5	0.453
YOLOX-Tiny	5.035	7.6	0.76	0.683	0.713	0.745	0.609	0.518
Vfnet	32.728	129	0.669	0.647	0.648	0.684	0.54	0.476
YOLOv8m	25.84	78.7	0.8046	0.6938	0.7367	0.7666	0.6857	0.6187
YOLOv9m	20.01	76.5	0.7537	0.6767	0.7055	0.7505	0.6638	0.604
YOLOv10m	15.32	58.9	0.7861	0.6821	0.7222	0.7718	0.6769	0.5994
YOLOv11m	20.04	67.7	0.7687	0.734	0.7463	0.7851	0.6747	0.6241
YOLOv26m	20.35	67.9	0.7271	0.699	0.7107	0.7498	0.6502	0.5947
Hyper-YOLO	13.51	33.8	0.7878	0.7186	0.7424	0.7535	0.6772	0.6059
GBR-DETR	22.13	59.4	0.8214	0.7291	0.7693	0.7891	0.6852	0.6298

**Table 7:** Edge device deployment and environment configuration.

Parameter	Setting
Deployment environment	NVIDIA Jetson Orin Nano Developer Kit
Operating system	Ubuntu 22.04 LTS (64-bit)
Jetpack version	Jetpack 6.2.1
Memory	8 GB LPDDR5
Processor	6-core Arm Cortex-A78AE v8.2 64-bit CPU
GPU	NVIDIA Ampere architecture with 1024 CUDA cores
Python	3.10.12
CUDA	12.6
cuDNN	9.3.0
TensorRT	10.3.0.26

**Table 8:** Performance of GBR-DETR and RT-DETR on the PlantDoc dataset.

Model	Precision	Recall	mAP_50_	mAP_75_	mAP_50–95_
RT-DETR	0.6001	0.5169	0.4649	0.4072	0.3566
GBR-DETR	0.6161	0.4947	0.486	0.4456	0.3847

## Data Availability

The code and dataset used in this study are publicly available at the following GitHub repository: https://github.com/zhuojiaxiong6/DETR (accessed on 29 April 2026). This repository contains the implementation of GBR-DETR, a Detection Transformer variant developed for detecting tomato leaf diseases and pests. All relevant training scripts, configuration files, and instructions for dataset usage are provided in the repository.

## Abbreviations

The following abbreviations are used in this manuscript:

ADown	Advanced Downsampling
AP	Average Precision
ATSS	Adaptive Training Sample Selection
BCPN	Bidirectional Context Pyramid Network
CFB	Core Feature Block
CNN	Convolutional Neural Network
Conv	Convolution
CPU	Central Processing Unit
CUDA	Compute Unified Device Architecture
cuDNN	CUDA Deep Neural Network library
DETR	Detection Transformer
DINO	DETR with Improved deNoising anchOr boxes
DWConv	Depthwise Convolution
ESFM	Edge–Semantic Fusion Module
F1	F1 score
Faster R-CNN	Faster Region-based Convolutional Neural Network
FP16	Half-precision (16-bit) Floating-Point
FP32	Single-precision (32-bit) Floating-Point
FPS	Frames Per Second
GBR-DETR	Gradient-aware Bidirectional Retentive Detection Transformer
GFL	Generalized Focal Loss
GFLOPs	Giga Floating-point Operations per second
GPG	Gradient Pyramid Generator
GPU	Graphics Processing Unit
Grad-CAM	Gradient-weighted Class Activation Mapping
HDR	High Dynamic Range
HW	Height × Width
IoU	Intersection over Union
JPEG	Joint Photographic Experts Group
JSON	JavaScript Object Notation
LPDDR5	Low-Power Double Data Rate 5
LTS	Long-Term Support
mAP	Mean Average Precision
MaxPool	Max Pooling
MGAT-Net	Multi-scale Gradient-Aware Transfer Network
M-TLD	Multi-scenario Tomato Leaf Disease (dataset)
NMS	Non-Maximum Suppression
ONNX	Open Neural Network Exchange
P	Precision
PANet	Path Aggregation Network
R	Recall
RelPos2d	Relative Position 2D encoding
RepC3	Reparameterized C3 module
ResNet	Residual Network
RetBlock	Retentive Block
RetNet	Retentive Network
RFAM	Retentive Feature Aggregation Module
RGB-D	Red-Green-Blue with Depth
RMT	Retentive networks Meet vision Transformers
RT-DETR	Real-Time Detection Transformer
SGD	Stochastic Gradient Descent
TensorRT	Tensor RunTime
TOOD	Task-aligned One-stage Object Detection
TYLCV	Tomato Yellow Leaf Curl Virus
VFNet	VarifocalNet
XAI	Explainable Artificial Intelligence
YOLO	You Only Look Once
